# CLDN6: From Traditional Barrier Function to Emerging Roles in Cancers

**DOI:** 10.3390/ijms222413416

**Published:** 2021-12-14

**Authors:** Huinan Qu, Qiu Jin, Chengshi Quan

**Affiliations:** The Key Laboratory of Pathobiology, Ministry of Education, College of Basic Medical Sciences, Jilin University, 126 Xinmin Avenue, Changchun 130021, China; quhn18@mails.jlu.edu.cn (H.Q.); jinqiu21@mails.jlu.edu.cn (Q.J.)

**Keywords:** tight junctions, CLDN6, barrier, cancer, signaling, applications

## Abstract

Claudins (CLDNs) are the most important tight junction proteins, which are mainly expressed in endothelial cells or epithelial cells in a tissue-specific manner. As a member of the CLDNs family, CLDN6 is highly expressed in fetal tissues such as the stomach, pancreas, lung, and kidney, but is not expressed in corresponding adult tissues. The expression of CLDN6 is regulated by a variety of factors, including but not limited to stimuli and transcription factors, DNA methylation, and post-translational modifications. CLDN6 has been found to have a key role in the formation of barriers, especially the lung epithelial barrier and the epidermal permeability barrier (EPB). Importantly, the roles of CLDN6 in cancers have gained focus and are being investigated in recent years. Strong evidence indicates that the altered expression of CLDN6 is linked to the development of various cancers. Malignant phenotypes of tumors affected by CLDN6 include proliferation and apoptosis, migration and invasion, and drug resistance, which are regulated by CLDN6-mediated key signaling pathways. Given the important role in tumors and its low or no expression in normal tissues, CLDN6 is an ideal target for tumor therapy. This review aims to provide an overview of the structure and regulation of CLDN6, and its traditional barrier function, with a special emphasis on its emerging roles in cancers, including its impact on the malignant phenotypes, signal-modulating effects, the prognosis of tumor patients, and clinical applications in cancers.

## 1. Introduction

Tight junctions (TJs) were first found at the apical side of epithelial cells by electron microscopy in the 20th century [1]. TJs are essential for epithelial and endothelial to establish a barrier between different tissue compartments to regulate the passage of molecules and ions between cells, which is the gate function of TJs [2]. Besides, TJs have a fence function that prevents the mixing of membrane lipids between the apical and basolateral membranes [3]. TJs consist of more than 40 proteins in two categories: transmembrane proteins and cytoplasmic actin-binding proteins. The former is responsible for the interactions between neighbor cells and constitutes the gate and fence function of TJs. The latter connects the TJs to the actin cytoskeleton. Transmembrane proteins consist of CLDNs, occludins (OCLDs), and junctional adhesion molecules (JAMs). Among them, CLDNs are the most important, because there is no paracellular barrier formed without CLDNs [4].

There are 26 (*human*) or 27 *(rodents*) CLDNs produced in epithelial and endothelial cells in a tissue-specific manner [5]. Many tissues express different CLDNs, which can interact with each other in both cis (intracellular) and trans (intercellular) interactions [6]. CLDN6 is homologous to CLDNs 1–5, 7–10, 14, 15, 17, and 19, all of which are classical while the others are less homologous non-classical CLDNs [7]. CLDN6 was identified from a differential display analysis of differentiating embryoid bodies (EBs) in 2001 [8], although its cDNA was isolated from an ectoderm-specific library in 1995 [9] and was amplified by RT-PCR from mouse kidney in 1999 [10]. The expression of CLDN6 is dynamically modulated by various factors. CLDN6 is expressed in fetal tissues including the stomach, pancreas, lung, and kidney, but not expressed in the corresponding adult tissue samples [11]. CLDN6 is one of the earliest proteins expressed in embryonic stem (ES) cells committed to the epithelial fate and a cell-surface-specific marker of human pluripotent stem cells (hPSCs) [12].

The traditional gate and fence function of CLDN6 is supported by the finding that homozygous mice overexpressing CLDN6 die within 48 h of birth due to a defective EPB [13]. Notably, Quan C [14] found that CLDN6 was preferentially expressed in mammary epithelial cells of Copenhagen rats, which were extremely resistant to mammary cancer development, compared with susceptible Buffalo. This important finding suggested that CLDN6 may be a suppressor gene in breast cancer and has stimulated a surge of research on the roles of CLDN6 in different cancers. The roles of CLDN6 in cancers cannot be explained by the barrier function, and they appear to be mediated by key signaling pathways. In this review, we first introduced the structure of CLDN6 and then described the regulation of CLDN6. Next, we summarized the traditional barrier function of CLDN6. Finally, we focused on the emerging roles of CLDN6 in cancers.

## 2. Structure

**Figure 1:**
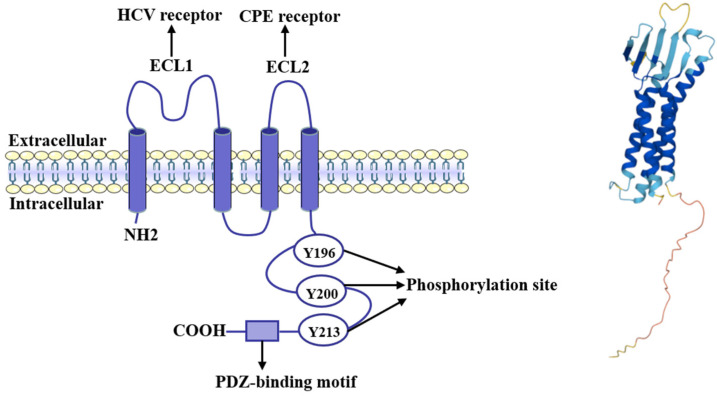
Schematic presentation and molecular diagram of CLDN6. The left shows the structure diagram of CLDN6. It contains four transmembrane domains, two extracellular ECL loops (ECL1 and ECL2), one amino-terminal, and one carboxy-terminal. The right shows the chemical structure of CLDN6 predicted by the AlphaFold [19,20].

In humans, the *CLDN6* gene is located at 16p13.3 and contains three exons. There are three GC boxes and three CpG islands in the regulator region of *CLDN6*. CLDN6 consists of 220 amino acids, including 20 different amino acids, the main components being 24 alanines, 21 glycines, 32 leucines, 19 serines, and 23 valines. The molecular formula of CLDN6 is C_1054_H_1682_N_268_O_291_S_16_ with a 23 kDa molecular weight. As shown in Figure 1, CLDN6 is a four-transmembrane protein with a short cytoplasmic N terminus and a C-terminal cytoplasmic domain, two extracellular domains (ECL1, which is larger and a smaller ECL2), and one short intracellular loop, which is consistent with other CLDNs. ECL1 and COOH-terminal PDZ-binding motif (PBM) are the typical domains of CLDNs. The ECL1 interdigitates between cells to form ion-selective pores. CLDN6 decreased chloride permeability when transfected into MDCK II cells [15]. Furthermore, the ECL1 of CLDN6 helps the Hepatitis C virus (HCV) enter into cells [16,17]. The PBM of CLDN6 can bind to proteins with PDZ domains such as ZO-1 to mediate cell signaling transduction, as will be discussed in Section 5.3. In addition, the ECL2 of CLDN6 contributes to the binding for clostridium perfringens enterotoxin (CPE) [18].

## 3. Regulation of CLDN6

### 3.1. Stimuli and Transcription Factors

The expression of CLDN6 is regulated by the degree of cell fusion and junctional maturation, as evidenced by the increase in expression with time and a high dependence upon plating density [8]. CLDN6 expression is also regulated by oxygen and estrogen, which are mediated by transcription factors such as HIF-1α [21,22] and Estrogen receptor α/β (Erα/β) [23,24], respectively. In the presence of tobacco smoke, CLDN6 is decreased in A549 and SAEC cells and mice lung tissues due to the interaction of HIF-1α and HIF-1α response element 1 (HRE1) at the *CLDN6* promoter [21]. However, HIF-1α promotes *CLDN6* transcription in hypoxic cultured breast cancer cells [22]. The two contrasting regulations of CLDN6 by HIF-1α may be related to the environment or tissue specificity, reflecting the complexity of protein regulation. Upon binding to estrogen, Erβ induces CLDN6 expression either by directly binding to the estrogen receptor response element (ERE) of CLDN6 or by binding to the transcription factor SP1 [24]. The key lung transcription factors thyroid transcription factor 1 (TTF-1), forkhead box A2 (FoxA2), and Gata-6 regulate the expression of CLDN6 from E15 to PN0 in mice lung tissues [25]. CLDN6 expression is increased in multidrug-resistant breast cancer cells and the relationship between CLDN6 and drug resistance will be discussed in Section 5.2.3. Here, we speculate that chemotherapeutic stimulation may also affect CLDN6 expression, but further validation is needed. As studies continue, more transcription factors regulating CLDN6 expression will be identified.

### 3.2. DNA Methylation

DNA methylation is a major epigenetic mechanism that leads to gene silencing by various mechanisms including, for example, methyl-DNA-binding proteins, changes in histone acetylation or inhibition of DNA-binding factors, as well as repositioning of nucleosomes thus preventing RNA polymerase II (Pol II) initiation [26,27]. Methylation of CLDN6 is associated with a decrease in mRNA expression in esophageal squamous cell carcinoma and breast cancer [28,29,30]. The methylation mechanism of CLDN6 has been studied mainly in breast cancer cells. DNA methylation of the CLDN6 promoter inhibits its expression through binding to MeCP2, deacetylating H3 and H4 [30]. At the transcriptional start site of CLDN6, Pol II is stalled and phosphorylated at ser2, which is relieved after treatment with demethylating agents such as Azacytidine, and the mRNA expression of CLDN6 is upregulated. Additionally, lymphoid-specific helicase (LSH), a member of the chromatin remodeling family, increases the DNA methylation and Pol II stalling of CLDN6 by enhancing the binding of DNMT3b to CLDN6 [31]. Lu Y et al. [32] found that SMAD2 inactivation promotes CLDN6 expression through repressing DNMT1-mediated the DNA methylation of CLDN6. Overall, DNA methylation is a well-explored regulator of CLDN6 expression in breast cancer cells, but whether this regulation is universal requires further investigation.

In addition, epigenetic modifications such as DNA methylation are regulated by microRNAs. It is found that miR-7 and miR-218 overexpression significantly decrease the DNA methylation and increase acetylation of H3 in the promoter region of CLDN6, which is associated with HoxB3 [33]. However, the mechanisms by which microRNAs regulate CLDN6 epigenetics and whether they can directly influence CLDN6 expression needs further investigation.

### 3.3. Posttranslational Modifications

Posttranslational modifications such as phosphorylation, ubiquitination, glycosylation, and palmitoylation are processes affecting the conformation, stability, trafficking, and function of proteins. The intracellular segment of CLDN6 contains several potential phosphorylation sites. Tyr-196/200 are phosphorylated by Src-family kinases (SFKs), and strong binding of SFKs to CLDN6-p-Tyr-196/200 fully activates SFKs, resulting in stimulating the downstream PI3K/AKT pathway [34]. Tyr-213 is another site shown to be phosphorylated, which is mediated by EphA7 (one of the Eph receptor tyrosine kinases family), reducing the distribution of CLDN6 at the cell surface [35]. Additionally, Rodenburg et al. [36] found that CLDN6 has four dominant palmitoylations sites by native mass spectrometry, but which palmitoyltransferase regulates CLDN6 palmitoylation needs to be further explored. Additional post-translational modifications on CLDN6 deserve further exploration, which will be essential for understanding the expression and translocation of CLDN6.

## 4. Traditional Barrier Function

CLDN6 has the traditional functions of tight junctions: permeability regulation and barrier formation. As for the permeability, CLDN6 is essential for the regulation of chloride permeability in the proximal tubules of the neonatal kidney [15] and the regulation of ion transport in the epithelium of the lymphatic sac in the inner ear [37,38]. In terms of barriers, CLDN6 is important in maintaining the lung epithelial barrier. Besides, CLDN6 is mainly involved in the formation of EPB in premature birth. Here, we introduced the traditional barrier function of CLDN6 in detail.

### 4.1. The Lung Epithelial Barrier

TJs are thought to be protective entries against epithelial inflammation and infection, and increased epithelial permeability contributes to inflammation in many respiratory diseases [39]. The change of CLDNs composition is regarded as a disturbance in barrier function, an important step that leads to smoke-induced lung disease. CLDN6 is implicated in maintaining the lung epithelial barrier and ameliorates inflammation of the respiratory barrier.

CLDN6 expression is inhibited by cigarette smoke and decreased oxygen tension via HIF-1α, culminating in barrier perturbation [21]. CLDN6 overexpression impacted secondhand tobacco smoke (SHS)-induced inflammation in the lung by reducing total bronchoalveolar lavage fluid (BALF) protein, cells, and secreted pro-inflammatory cytokines [40]. Similarly, CLDN6 decreases fine diesel particulate matter (DPM)-induced pulmonary inflammation in the same way [41]. Taken together, epithelial barriers organized by CLDN6 reduce cigarette or diesel-induced inflammation in the lung. However, further studies are needed to fully elucidate the distinct roles of CLDN6 and other tight junction proteins in hopes of discovering therapeutically beneficial targets in the exposed lung epithelial barrier.

### 4.2. The Epidermal Permeability Barrier (EPB)

Preterm birth is a major global health problem that causes a large number of infant deaths, many of which can be attributed to complications of immature EPB [42]. An integral EPB in premature birth is crucial for maintaining temperature stability, preventing microbial infection through the skin. CLDN6 expression has a key role in EPB formation.

Homozygous mice overexpression of Cldn6 resulted in a nonintact EPB and died within 2 days of birth [13]. However, heterozygous mice overexpressing Cldn6 (Inv-Cldn6) are also born with an incomplete EPB, but the mice continue to form a barrier and reach normal levels by postnatal day 12, allowing them to survive to adulthood [43]. This suggests that CLDN6 expression has a key role in EPB formation and that its overexpression leads to EPB defects while moderate expression is associated with the intact establishment of EPB.

Several studies have shown that the tail domain of CLDN6 is essential for its facilitation of EPB formation. Mice overexpressing Cldn6 lacking its entire tail domain (Inv-Cldn6-CΔ187) still form a functional barrier; however, lifelong abnormal epidermal hyperproliferation is observed after birth [44]. The skin of 2-week-old mice overexpressing a shorter deletion in the cytoplasmic tail domain of Cldn6 (Inv-Cldn6-CΔ196) have striking histological abnormalities compared with the wild-type, as evidenced by epidermal hyperplasia and hyperkeratosis, which is similar to the skin of Inv-Cldn6 and Inv-Cldn6-CΔ187 mice [45]. Mice expressing Cldn6 with a shorter tail deletion (Inv-Cldn6-CΔ206) possess a distinct developmental defect in epidermal differentiation resulting in EPB formation delays [42]. The above mutants of CLDN6 lead to its cytoplasmic localization while affecting the expression and localization of other CLDNs, such as CLDN5, 8, 10, 11, 12, and 18, emphasizing the importance of the CLDN6 tail domain for membrane targeting and suggesting that the CLDN6 tail domain is essential for CLDNs homeostasis and EPB formation.

## 5. Emerging Roles in Cancers

The loss of cell-to-cell junctions is one of the important factors in cellular transformation and tumorigenesis [46]. Notably, more than simply being static components that establish tight junctions, CLDNs are also cellular signaling components to receive environmental cues and transmit signals within the cell, which explains their involvement in cancer growth and progression [47]. As a member of CLDNs, CLDN6 is also associated with tumor initiation and progression in a range of cancers.

### 5.1. The Expression of CLDN6 in Cancers

The expression pattern of CLDNs varies among cancer types and the expression of CLDN6 has been explored from the perspective of pan-cancer analysis, in which CLDN6 was found significantly upregulated in 20 types of cancers while it is downregulated in glioblastoma multiforme (GBM), kidney chromophobe (KICH), kidney renal clear cell carcinoma (KIRC), acute myeloid leukemia (LAML), and brain lower-grade glioma (LGG) [48].

In addition to the pan-cancer analysis, Tetsuo Ushiku et al. used TMA (containing 860 tumors) to detect and analyze CLDN6 expression in germ cell tumors and visceral carcinomas by IHC. The results showed that CLDN6 was more highly expressed in germ cell tumors (28/28, 100%) and less expressed in non-germ cell tumors (64/832, 8%) [49]. We summarized the results of CLDN6 expression in different tumors in this study with other IHC results about CLDN6 in tumors in Table 1. The comparison revealed that the expression of CLDN6 in the same tumor may not be identical or even contradictory in different studies. For example, Tetsuo Ushiku et al. [49] found a positive rate of 0% for CLDN6, while Yan Lu et al. [50] found a positive expression rate of 79.2% in hepatocellular carcinoma. This may be related to intratumor heterogeneity, the number of cases, different experimental methods, or different manufacturers of antibodies, and further validation is needed as to whether CLDN6 can be a biomarker for identifying certain tumors.

### 5.2. The Malignant Phenotypes of CLDN6 Affected in Cancers

Studies have shown that CLDN6 inhibits the development and progression in some cancers such as breast cancer [22,24,61,62,63], cervical cancer [56], and meningioma [64]. In hepatocellular cancer [50,65,66], gastric cancer [52,67], endometrial cancer [68,69], and ovarian cancer [70], CLDN6 has the opposite roles. The oncogenic role or conversely tumor-suppressive effect of CLDN6 in different types of cancer is shown in Table 2. The roles of CLDN6 in proliferation and apoptosis, migration and invasion, and drug resistance will be discussed in the following sections.

#### 5.2.1. Proliferation and Apoptosis

The relationship between CLDN6 and tumor cell proliferation and apoptosis is confirmed by gain- and loss-of-function experiments in a variety of tumor cells. In MCF-7 breast cancer cells, CLDN6 overexpression inhibits proliferation and anchorage-independent growth [61] and induces apoptosis via ASK1-P38/JNK signaling in MCF-7 cells [63]. Besides, suppression of CLDN6 in MCF-7 or HBL-100 cells results in increased cell proliferation, and CLDN6 exerts this role in HBL-100 cells in association with P38/MAPK signaling activation [62,73]. Similarly, CLDN6 overexpression in cervical carcinoma cells inhibits tumor growth in vitro and in vivo [56]. However, CLDN6 promotes the proliferation of human hepatocellular carcinoma cells [50,65]. CLDN6 also acts as a tumor promoter to increase cell proliferation in gastric cancer [52]. Knockdown of CLDN6 significantly inhibited HEC-1B endometrial cancer cell proliferation via PI3K/Akt/mTOR signaling pathway [68]. In summary, CLDN6 is involved in the regulation of cancer cell proliferation and apoptosis through different pathways (Table 2), which results in pro- or anti-cancer effects of CLDN6 in different cancers.

#### 5.2.2. Migration and Invasion

The molecular mechanisms of cell migration and invasion are complicated, involving epithelial mesenchymal transition (EMT) [74], matrix metalloproteinases (MMPs) [75], autophagy [76], tumor microenvironment (TME) [77], and so on. Many studies have shown that CLDN6 affects tumor cell migration and invasion, but there is a paradox in that CLDN6 both inhibits and promotes migration and invasion (Table 2).

EMT is one of the important mechanisms by which CLDN6 affects migration and invasion. In gastric cancer cells, CLDN6 interacted with LATS1/2 to reduce the phosphorylation of YAP, resulting in the level of YAP increasing in the nucleus, which interacts with snail1 to bind to the promoter of EMT related genes to enhance the migration and invasion abilities [52]. CLDN6 silencing inhibits the migration and invasion abilities of HCC cells, which may be dependent on its repression of EMT [50].

MMPs promote the migration and invasion of tumor cells by mediating the degradation of the extracellular matrix. In ovarian papillary serous carcinomas, CLDN6 and MMP-2 are up-regulated detected by IHC, but the result can’t prove a causative connection [54]. The regulation of MMP2 by CLDN6 and the role of both in cell migration and invasion are demonstrated in gastric cancer cells: CLDN6 increases the activity of MMP-2 to enhance cell migration and invasion by upregulating the expression CLDN1 [67]. Gene silencing of CLDN6 enhances cell migration and invasion accompanied by increased MMP-2 activity in human breast epithelium cell line HBL-100 [62].

Autophagy produces fatty acids, amino acids, and nucleotides by degrading damaged proteins or organelles to enable cells to maintain adequate metabolism and survive under conditions of nutrient stress. In addition, autophagy has a key role in inducing a state of response to hypoxia in EMT and stem cells, and in regulating signals such as TGF-β, suggesting that autophagy is significant in tumor metastasis [76]. When observing the effect of CLDN6 on cell tight junctions by transmission electron microscopy, a large number of autophagic vesicles were unexpectedly observed. The authors also showed that CLDN6 increased the expression of several autophagy-related proteins such as BECLIN1, ATG5, ATG16, and LC3-II. They subsequently demonstrated that the inhibitory effect of ERβ on breast cancer metastasis was dependent on CLDN6-mediated autophagy [24]. However, whether the effect of CLDN6 on migration and invasion in other tumors is related to autophagy needs to be further investigated.

Rapid tumor growth and intratumoral hypoxia caused by abnormal tumor vascularity leads to a significantly increased risk of breast cancer metastasis which is mainly mediated by HIF-1α [78]. Under hypoxia, CLDN6 is transcriptionally upregulated by HIF-1α and inhibits breast cancer cells migration and invasion by combining and decreasing β-catenin in the cytoplasm, then reducing the transcriptional regulation of SENP1 by β-catenin, which prevents the deSUMOylation of HIF-1α and leads to HIF-1α degradation, suggesting there is a negative feedback loop between HIF-1α and CLDN6 [22].

The above studies have demonstrated that CLDN6 plays an important role in the migration and invasion of tumor cells in vitro. In breast cancers with low CLDN6 expression, CLDN6 overexpression inhibits tumor cell migration and invasion, while in cancers such as hepatocellular cancer with high CLDN6 expression, CLDN6 acts as a promoter of migration and invasion. Further mechanistic studies are needed to determine the context-dependent mechanisms. It is worth noting that several studies have used nude mice to establish tumor metastasis models, such as the liver metastasis model by injecting gastric cancer cells into the spleen [52] and lung metastasis model by tail vein injection of breast cancer cells [22,24] to observe the effect of CLDN6 on tumor metastasis, and obtained results consistent with the in vitro experiments, which profoundly demonstrated the effect of CLDN6 on tumor metastasis.

#### 5.2.3. Drug Resistance

CLDNs such as CLDN1, 2, 3, 7, and 11 are known to modulate drug resistance in cancers [79]. Yang et al. [71] found that CLDN6 was highly expressed in the MCF-7 multidrug-resistant (MDR) cells, and CLDN6 upregulated the expression of glutathione S-transferase-p1 (GSTP1) to promote chemoresistance of MCF-7 cells. However, in triple-negative breast cancer cells MDA-MB-231, GSTP1 is lost, CLDN6 enhances chemoresistance to ADM via activating the AF-6/ERK signaling pathway and up-regulating cancer stem cell characters [72]. Cancer cells with high plasticity can undergo phenotypic switching to a drug-resistant state to avoid drug toxicity, which made tumor plasticity an important mechanism of drug resistance and tumor relapse. CLDN6 activates the Hippo signaling and induces a phenotypic shift from hepatic lineage to biliary lineage, which makes HCC cells more resistant to sorafenib [66]. These findings highlight the significance of CLDN6 as a novel target in improving the efficacy of cancer therapy.

### 5.3. Signaling Pathways Involved in CLDN6

CLDN6 not only maintains tissue integrity but also possesses signaling properties that contribute to diverse cellular events such as cell proliferation, apoptosis, migration, and invasion as discussed above. The mechanism of how CLDN6 transmits signaling is not very clear, but there are two theories currently. On the one hand, CLDN6 binds to PDZ-containing proteins through its PBM to mediate signaling transduction. For example, CLDN6 combines with PDZ-containing protein ZO-1, and then ZO-1, UVRAG, and BECLIN1 form complexes to regulate autophagosome formation in breast cancer cells [24]. In MDA-MB-231 breast cancer cells, CLDN6 binds to another PDZ-containing protein AF-6 to inhibit its downstream ERK activation [72]. CLDN6 is found upregulated and interacted with ZO-2/YAP1, which activates the Hippo signaling in HCC cells [66]. In gastric cancer cells, CLDN6 interacted with LATS1/2 and decreased the conversion of LATS into p-LATS, thereby inhibiting the Hippo signaling pathway. But the mechanisms of how CLDN6 binds to LATS1/2 are not very clear, may be associated with ZO-1 [52].

On the other hand, CLDN6 activates a series of kinases depending on ECL2 and Tyr-196/200 to transmit cell adhesion signals to the nucleus and regulate gene expression. The retinoic acid receptors (RARs), one member of the nuclear receptor superfamily, transcriptionally regulate the expression of many target genes. RARγ heterodimers can trigger epithelial differentiation, which is similar to CLDN6, so researchers tried to find the link between CLDN6 and RARγ. It was concluded that ECL2-dependent, CLDN6-mediated cell–cell adhesion recruits and activates the SFK/PI3K/AKT signaling pathway and stimulates the RARγ and ERα activities in F9 stem cells [34]. Also, CLDN6 regulates the PI3K/AKT/mTOR signaling pathway in HEC-1B endometrial carcinoma cells [68] and EGFR/AKT/mTOR signaling pathway in HepG2 cells [65], but the mechanisms of how CLDN6 affects the signaling pathway in both HEC-1B and HepG2 cells remain to be verified.

### 5.4. CLDN6 and Patient Prognosis

CLDN6 expression is associated with the patient prognosis of a variety of tumors. High expression of CLDN6 is associated with worse overall survival (OS), disease-specific survival (DSS), and progression-free interval (PFI) in adrenocortical carcinoma (ACC), bladder urothelial carcinoma (BLCA), stomach adenocarcinoma (STAD), and uterine corpus endometrial carcinoma (UCEC), especially in different clinical subgroups of UCEC, which were analyzed by integrative multiple omics in pan-cancer [48].

In addition, several studies have analyzed the relationship between CLDN6 and tumor prognosis. In NSCLC, high CLDN6 expression is associated with a shorter OS of patients in univariate and multivariate [51,80]. In endometrial cancer, high CLDN6 expression is an independent prognostic marker for a poor OS of patients [55]. In gastric cancer, an OS analysis using the Kaplan–Meier plotter indicates that patients with high CLDN6 expression have a poor prognosis [52]. Consistently, Kohmoto T et al. [53] showed that gastric cancer patients with higher CLDN6 had a worse OS. However, Gao F et al. [81] found that the survival rate of patients was significantly higher in the CLDN6 high-expression group than that in the CLDN6 low-expression group. In breast cancer, higher CLDN6 expression is significantly related to a longer OS and disease-free survival (DFS) of patients analyzed by the Kaplan–Meier plotter [24]. As for colorectal tumors, low CLDN6 expression in CMS2 (epithelial and canonical) subtype is associated with a longer OS and PFS [82]. Thus, abnormal expression of CLDN6 in certain tumors is closely associated with patient prognosis, but it seems that more research is needed.

### 5.5. Clinical Applications of CLDN6

**Figure 2:**
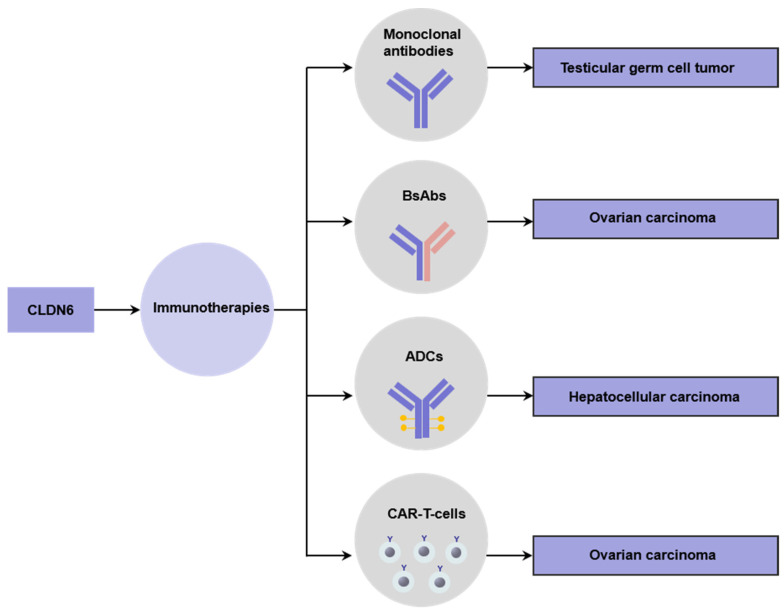
Clinical application of CLDN6 immunotherapy. CLDN6 is applied in monoclonal antibodies for testicular germ cell tumor, BsAbs for ovarian carcinoma, ADCs for hepatocellular carcinoma, and CAR-T-cells therapy for ovarian carcinoma.

CPE is a single chain 319 amino-acid axin that causes rapid cell lysis [83]. CLDN6 is a target for CPE, which made a possibility for it to act as a targeted therapy for cancers that expressed CLDN6 [18]. CLDN6 expression is aberrantly activated in various cancer types such as mentioned in Section 5.1, so CLDN6 is an ideal target for antibody approaches of high potency. CLDN6 is studied to be used for emerging immunotherapies to treat cancers including monoclonal antibodies, bispecific antibodies (BsAbs), antibody–drug conjugates (ADCs), and chimeric antigen receptor (CAR) T-cell therapy (Figure 2).

Using monoclonal antibodies against CLDN6 can repress tumor growth and extend the lifespan of testicular germ cell tumors (TGCTs) patients [84].

BsAbs contain two different antigen-binding sites in one molecule, which can enhance cytotoxicity by directing specific effectors of the immune system to target tumor cells [85]. 6PHU3, a T-cell-engaging bispecific single-chain molecule (bi-(scFv)2) with anti-CD3/anti-CLDN6 specificities, is highly specific and efficient for the treatment of CLDN6-positive solid cancers [86].

ADCs are biologic drugs consisting of monoclonal antibodies conjugated to biologically active drugs through chemical linkers, which combine the high specificity and targeting of monoclonal antibody drugs with the high efficiency of small molecule drugs in killing cancer cells, being one of the fastest-growing fields in cancer therapy [87]. Anti-CLDN6 monoclonal antibody conjugated with cytotoxic agent (Mertansine) DM1 (CLDN6-DM1) was developed and showed preclinical antitumor activity in HCC treatment [66].

CAR-T cell therapy represents a major advancement in personalized cancer treatment. One of the challenges of CAR-T cell therapy for solid tumors is limited cancer-specific targets. CLDN6 is an ideal expression profile for CAR-T cells targeting as a strictly oncofetal cell surface antigen [11].

## 6. Conclusions and Perspectives

As a member of the tight junction protein CLDNs family, CLDN6 has two extracellular loops, one amino-terminal and one carboxy-terminal. CLDN6 is regulated by stimuli and transcription factors, DNA methylation, phosphorylation, and palmitoylation. CLDN6 has a traditional barrier function in regulating the lung epithelial barrier and the EPB. Importantly, CLDN6 has emerging roles in cancers. The PBM at the carboxyl terminus and the second extracellular loop mediate the signaling effects of CLDN6, which is an important mechanism by which CLDN6 affects tumor cell proliferation and apoptosis, migration and invasion, and drug resistance. Since CLDN6 is not or lowly expressed in most adult tissues, vaccines and drugs targeting CLDN6 are ideal for tumor prevention and treatment in solid tumors with high CLDN6 expression.

Although we have the above understanding of CLDN6, this is not enough. The following is an attempt to summarize the unknown and future directions of research on CLDN6: (1) the reasons why CLDN6 is lowly or not expressed in most adult tissues but highly expressed in a few tissues, such as the breast; (2) CLDN6 is highly expressed in some tumors and lowly in others; (3) whether the signaling pathways involved in CLDN6 regulation in tumors are tissue or cell-specific, in other words, whether CLDN6 has a classical signaling pathway; (4) whether posttranslational modifications of CLDN6 are involved in its regulation of tumors; (5) the degradation pathway and mechanism of CLDN6. We believe that CLDN6 will have an even more important role in the prevention, diagnosis, and treatment of cancers with further research.

## Figures and Tables

**Table 1:** Expression of CLDN6 in different cancer types.

Type of Cancer	No. of Cases	Positive Cases (%)	References
Germ cell tumors	28	28 (100)	[49]
Non-small-cell lung cancer	119	13 (10.9)	[49]
	355	23 (6.5)	[51]
Breast cancer	70	1 (1.4)	[49]
Oesophagus Squamous cell carcinoma	19	0 (0)	[49]
Gastric cancer	72	7 (9.7)	[49]
	494	255 (51.6)	[52]
	208	28 (13.5)	[53]
Hepatocellular Carcinoma	48	0 (0)	[49]
	48	38 (79.2)	[50]
Pancreas Adenocarcinoma	48	1 (2.1)	[49]
Large intestine Adenocarcinoma	96	0 (0)	[49]
Ovarian carcinoma	144	35 (24.3)	[49]
	62	34 (54.8)	[54]
Endometrial Cancer	24	5 (20.8)	[49]
	173	47 (32.9)	[55]
Cervical carcinoma	56	16 (28.6)	[56]
Kidney carcinoma	48	0 (0)	[49]
Bladder carcinoma	25	2 (8)	[49]
Prostate Adenocarcinoma	47	0 (0)	[49]
Skin tumors	24	0 (0)	[49]
Atypical Teratoid/Rhabdoid Tumors	7	7 (100)	[57]
	59	17 (28.8)	[58]
	31	12 (38.7)	[59]
Meningioma	10	1 (10)	[59]
Myxofibrosarcoma	61	39 (63.9)	[58,60]

**Table 2:** The function of CLDN6 in different cancer types.

Type of Cancer	Activity	Function	Signaling Components	References
Breast cancer	Tumor Suppressor	Inhibits proliferation and Induces apoptosis	P38/MAPK signaling	[61,62]
		Inhibits invasive and migratory abilities	ASK1/P38/JNK signaling	[63]
			MMP2	[62]
			BECLIN1-dependent autophagy	[24]
			β-catenin/SENP1/HIF-1α	[22]
	Tumor Promoter	Promotes drug resistance	GSTP1	[71]
			AF-6/ERKs signaling	[72]
Cervical cancer	Tumor Suppressor	Inhibits proliferation and Induces apoptosis	-	[56]
Meningioma	Tumor Suppressor	Inhibits invasive and migratory abilities	MMP-2, MMP-9, vimentin, and N-cadherin	[64]
Hepatocellular cancer	Tumor Promoter	Promotes proliferation	EGFR/AKT/mTOR signaling	[65]
		Promotes migration and invasion abilities	E-cadherin, N-cadherin and Vimentin	[50]
			EGFR/AKT/mTOR signaling	[65]
		Promotes drug resistance	ZO-2/YAP1	[66]
Gastric cancer	Tumor Promoter	Promotes proliferation	-	[52]
		Promotes migration and invasion abilities	YAP1/SNAIL axis	[52]
			CLDN1/MMP2 axis	[67]
Endometrial cancer	Tumor Promoter	Promotes proliferation	PI3K/AKT/mTOR signaling	[68]
			SFK/PI3K/AKT/ERα signaling	[69]
		Promotes migration and invasion abilities	PI3K/AKT/mTOR signaling	[68]
			SFK/PI3K/AKT/ERα signaling	[69]
Ovarian cancer	-	Immune cell infiltration	B cells, CD8+T cells, effector memory CD4+T cells, M1 macrophages, and myeloid dendritic cells	[70]
